# Cross-cultural adaptation of the everyday cognition scale (M-ECog) in older Mexican adults with cognitive impairment

**DOI:** 10.1590/1980-5764-DN-2023-0011

**Published:** 2023-10-23

**Authors:** Sara Gloria Aguilar-Navarro, Brenda Lorena Pillajo Sánchez, Lidia Antonia Gutiérrez Gutiérrez, Natalia Arias-Trejo, Yakeel T. Quiroz, Alberto José Mimenza Alvarado

**Affiliations:** 1 Instituto Nacional de Ciencias Médicas y Nutrición Salvador Zubirán, Geriatrics Department, Mexico City, Mexico.; 2 Instituto Nacional de Ciencias Médicas y Nutrición Salvador Zubirán, Department of Neurology and Psychiatry, Mexico City, Mexico.; 3 Universidad Nacional Autónoma de México, Faculty of Psychology, Mexico City, Mexico.; 4 Harvard Medical School, Massachusetts General Hospital, Department of Neurology and Psychiatry, Boston, MA, USA.

**Keywords:** Cognitive Dysfunction, Dementia, Questionnaires, Elderly, Memory, Disfunção Cognitiva, Demência, Questionários, Idoso, Memória

## Abstract

The Everyday Cognition (ECog) scale was created to evaluate the functional abilities of older adults across a wide range of abilities between normal aging and dementia. ECog screens cognitive alterations such as subjective cognitive decline (SCD) and mild cognitive impairment (MCI). This early recognition is done by the measurement of the ability to perform the activities of daily living (ADLs).

**Objective:**

To establish the cross-cultural adaptation, validity, and reliability of the ECog Mexican version (M-ECog) in participants with: SCD, MCI, and dementia coming from a memory clinic.

**Methods:**

There were 200 patients and their respective informants in a memory clinic of a third level hospital in Mexico City. Four groups were studied: 50 cognitively healthy (CH), 50 SCD, 50 MCI, and 50 dementia. The clinical evaluation included: sociodemographic and health characteristics, cognitive status by the Mini-Mental State Evaluation (MMSE) and Montreal Cognitive Evaluation Spanish version (MoCA-E), and caregiver information (informants) about the difficulty in ADLs as well as the ECog Spanish version (M-ECog).

**Results:**

The M-ECog was significantly correlated with MMSE, MoCA-E, and ADLs. It showed the ability to discriminate the different cognitive declines (Cronbach’s alpha 0.881). The intra-class correlation coefficient was 0.877 (95% confidence interval — CI, 0.850–0.902; p<0.001). The patient’s group area under curve (AUC) of M-ECog for SCD was 0.70 (95%CI 0.58–0.82, p<0.005), for MCI it was 0.94 (95%CI 0.89–0.99, p<0.001) and for dementia 0.86 (95%CI 0.79–0.92, p<0.001).

**Conclusion:**

The M-ECog scale proves to be valid and reliable for measuring everyday abilities mediated by cognition. It is self-applicable without requiring extensive prior formation. It is useful to screen for SCD and MCI in older Mexican adults.

## RESUMO

A escala Cognição Cotidiana (ECog) foi criada para avaliar as habilidades funcionais de idosos em uma ampla gama de habilidades entre o envelhecimento normal e a demência. O ECog rastreia alterações cognitivas como declínio cognitivo subjetivo (DCS) e comprometimento cognitivo leve (CCL). Esse reconhecimento precoce é feito pela mensuração da capacidade de realizar as atividades de vida diária (AVD).

**Objetivo:**

Estabelecer a adaptação transcultural, validade e confiabilidade da versão mexicana do ECog (M-ECog) em participantes com: SCD, MCI e demência provenientes de uma clínica de memória.

**Métodos:**

Foram 200 pacientes e seus respectivos informantes em uma clínica de memória de um hospital de terceiro nível na Cidade do México. Quatro grupos foram estudados: 50 cognitivamente saudáveis (CH), 50 SCD, 50 MCI e 50 com demência. A avaliação clínica incluiu: características sociodemográficas e de saúde, estado cognitivo pelo Mini-Mental State Evaluation (MMSE) e Montreal Cognitive Evaluation versão em espanhol (MoCA-E), bem como informações do cuidador (informantes) sobre a dificuldade nas AVD e o ECog versão em espanhol (M-ECog).

**Resultados:**

O M-ECog foi significativamente correlacionado com MMSE, MoCA-E e AVD. Mostrou capacidade de discriminar os diferentes declínios cognitivos (alfa de Cronbach 0,881). O coeficiente de correlação intraclasse foi de 0,877 (intervalo de confiança de 95% — IC95%, 0,850–0,902; p<0,001). A AUC do grupo do paciente de M-ECog para SCD foi de 0,70 (IC95% 0,58–0,82, p<0,005), para MCI foi de 0,94 (IC95% 0,89–0,99, p<0,001) e para demência foi de 0,86 (IC95% 0,79–0,92, p<0,001).

**Conclusão:**

A escala M-ECog mostra-se válida e confiável para medir habilidades cotidianas mediadas pela cognição. É autoaplicável sem exigir extensa formação prévia. É útil para rastrear MSC e MCI em adultos mexicanos mais velhos.

## INTRODUCTION

The ability to perform activities of daily living (ADLs) is one of the first signals that we have to make the early identification of cognitive impairment^1^. Currently, there are few scales available to systematically review cognition-mediated milder functioning problems. Daily functioning deficiencies are those that ultimately affect both individual independence and autonomy, diminishing quality of life, increasing the caregiver’s burden, increasing and contributing to financial costs related to the disease^2^. Thus, it is essential that the patient’s cognitive information be corroborated by an informant (caregivers, relatives, spouses, etc.). Therefore, in the medical evaluation, the individual perspectives of patients with an early cognitive impairment through mild to moderate dementia and their informants should be solicited because caregiving is inherently a dyadic process^3^.

At present, the tools available to assess the impact on functional status associated with cognition are scarce. ADLs alteration is an important criterion to distinguish mild cognitive impairment from dementia^4^, with instrumental activities being those affected first by cognition followed by basic activities of daily living^5^. For example, if two patients have a similar level of cognitive impairment, the one with no ADLs impairment is diagnosed with mild cognitive impairment (MCI), while the other with ADLs impairment is diagnosed with dementia^6^. However, recent studies have reported that mild levels of daily living dysfunction are often seen even at the stage of mild cognitive impairment. Incipient cognitive disorders are mistakenly considered a normal part of aging, but worldwide working groups consider early changes that must be detected in a timely manner to prevent patients from arriving with late diagnoses of dementia.

Thus, subjective cognitive decline (SCD) is the self-experienced persistent decline of cognitive ability compared to a previous normal state, not related to acute events, and with a normal performance on standardized cognitive tests (adjusted for age, sex, and education)^7^ and which in turn precedes MCI; a condition that is frequently underdiagnosed in clinical practice^8^. The SCD and MCI must be evaluated objectively through a comprehensive cognitive examination and ideally confirmed by an informant^9^. This is because, when it is corroborated by an informant, the risk of progression is greater compared to when only self-reported complaints are available. In addition, a close follow-up with a periodic clinical and neuropsychological evaluation is recommended with the aim of screening for cognitive decline^10^.

The Everyday Cognition (ECog) scale was developed in Canada in 2008, in response to some of the limitations of existing cognitive screening instruments, since many of the questionnaires do not include information from the informants^11^. ECog has two versions, one for the patient’s self-rating and another for the patient’s rating by an informant^12^. The original version of ECog consists of 39 items, with six cognitive domains: memory, language, visuospatial and perceptual skills, planning, organization, and divided attention. Each functional domain subjectively assesses very early and subtle alterations in cognitive abilities compared to their previous performance from ten years earlier. ECog does not have established cut-off points, but greater cognitive impairment is related to higher scores^13^.

The ECog has also been validated in several countries such as Spain^14^, Argentina^15^, and Korea^16^. These studies support its psychometric results (content, construction, convergent, divergent, internal, external, and reliability validity), which are acceptable and reproducible in different population groups. So far, Mexico’s literature does not report ECog validation studies. Therefore, the goal of the current study was to establish the cross-cultural adaptation, validity, and reliability of the ECog Mexican version (M-ECog) in participants with SCD, MCI, and dementia coming from a memory clinic.

## METHODS

### Participants

Cross-sectional and validation study including 200 participants aged over 60 and their respective caregivers as informants (total 400). Patients were recruited consecutively from May 1^st^ to November 1^st^, 2022, as they attended the memory clinic of a third-level hospital in Mexico. The sample was estimated with the goal of conducting a critical study of both diagnostic performance and validation by comparing M-ECog, Mini-Mental State Evaluation validated in Mexico (MMSE)^17^ and Montreal Cognitive Evaluation Spanish version (MoCA-E)^18^. Considering a moderate correlation, an alpha error equal to 5%, and a power of 80%, at least 50 patients per group were needed to test both diagnostic performance and the hypothesis to be validated^19^.

Cognitive status was determined according to current clinical criteria (Gold Standard), and four groups were established: 50 cognitively healthy (CH), 50 with SCD^7^, 50 with MCI (Diagnostic Criteria and Statistical Manual of Mental Disorders, version 5 [DSM-5])^20^, and 50 with dementia (Criteria of the National Institute on Aging-Alzheimer’s Association [NIA-AA] of 2011)^21^. The same evaluator (a certified geriatrician) made a clinical and cognitive evaluation that included: MMSE, MoCA-E, and ECog version translated into Spanish (M-ECog).

To assess the basic and instrumental ADLs (IADLs) we used two scales. First, the Katz scale^22^ which identifies a person’s degree of independence to accomplish basic activities of life that include six functions (bathing, dressing, toileting, transferring, continence, and feeding) and has a maximum score of 6 out of 6 points, meaning 6/6 total independence and any score lower than 6 indicating functional impairment (dependence). Second, we used the Lawton & Brody scale^23^ that assesses the person’s degree of independence to carry out instrumental activities of life, which include eight functions: the ability to use the telephone, go shopping, prepare food, take care of the house, wash clothes, use transport, and be responsible for medications and finances. This scale has a scoring system that awards 1 point for every activity properly done or 0 points if this is not the case. The maximum score is 8/8 (total independence) and any score lower than 8 indicates functional impairment (dependence).

For every group, inclusion criteria were specified:

- CH group: age ≥60 years, female or male, with normal MMSE and MoCA-E scores;
- SCD group^7^, people with SCD in the last five years that causes concern and is confirmed by an informant;
- MCI group, MMSE ≥18 points and MoCA-E ≥18 points, with Clinical Classification of Dementia — CDR^24^=0.5 and DSM-5 criteria for minor neurocognitive disorder;
- Dementia group, with MMSE and MoCA-E ≤17 points and a CDR of 1.0–2.0, positive DSM-5 and NIA-AA 2011 criteria^21^;
- Caregiver/informant, someone who has had contact with the patient ≥40 hours per week in the last six months, male or female, formal or informal, and who can provide information on the functional and cognitive status of the patient. The rest of the patients’ information was obtained from the medical record.

The exclusion criteria were no schooling (not knowing how to read or write), major depression (Geriatric Depression Scale — GDS > or equal to 6 points)^24^, decompensated metabolic and neurological pathologies, significant abuse of alcohol, substances, sedatives, antipsychotics and benzodiazepines that make neuropsychological tests impossible. For the informant, the exclusion criteria were the self-report of depression without treatment and/or the diagnosis of a neurocognitive disorder.

### Research tools

ECog is an updated version scale^25^ including activities that involve the use of technology and has 41 items evaluating six cognitive domains and global cognition: memory, language, visuospatial and perceptive abilities, planning, organization, and divided attention. Each item is rated using a Likert scale over 4 points: 1=better or no change; 2=occasionally worse; 3=consistently a little worse; 4=consistently much worse; 0=don’t know/not applicable^13^. Each functional domain is related to the corresponding most affected cognitive abilities through daily life. So, a higher cognitive impairment relates to a higher rating (max. 164 points). Both the patient and the informant (M-ECog) completed the ECog by self-report, and the Zarit questionnaire was also applied to the informant, which identifies the caregiver’s burden: a score greater than 46 corresponds to mild overload and scores greater than 55 indicate intense overload^26^.

### Procedures

The cross-cultural validation of the ECog was carried out by researchers from the Faculty of Psychology of the Universidad Nacional Autónoma de México (UNAM). It consisted of a corroboration of the content, construct, convergent, divergent, and discriminative validity of M-ECog. This was done by comparing M-ECog *vs*. MMSE and M-ECog *vs*. MoCA-E and adjusting some words and syntax of the instructions and sentences presented to Mexican Spanish to both the patient and the informant (Supplementary Material 1). The activities were reviewed with a scale of similar terminology from some regions of the country in small groups of elderly volunteers who attended their respective appointments at the memory clinic (M-ECog). In addition, the reliability of M-ECog was confirmed to the extent that this instrument systematically obtained the same results when used in the same situation on repeated occasions by the participants and their respective caregivers.

### Statistical analysis

The validity of this instrument content was already proved by the original author in 2008^13^. The group differences were examined using a one-way analysis of variance (ANOVA). Significant ANOVA results were followed by *post-hoc* comparisons using the Bonferroni correction. Pearson’s χ^2^ tests were performed for categorical variables. Internal consistency was evaluated using Cronbach’s alpha coefficient. Convergent validity was assessed by calculating the intra-class correlation coefficient (ICC) between M-ECog, MMSE, MoCA-E, and ADLs. A curve analysis of Receiver Operating Characteristic (ROC) was made, and it was also calculated an area under curve (AUC) to examine the ability of M-ECog to discriminate between CH and SCD, MCI and dementia in both patients and informants. Sensitivity (S), specificity (Sp), positive predictive value (PPV) and negative predictive value (NPV), and cut-off scores of the M-ECog (ECog-Mexico) were obtained by ROC curve analysis and ANOVA using the IBM Statistical Package for the Social Sciences — SPSS 21.0 program (Armonk, NY, USA).

For the cross-cultural adaptation of the ECog scale, a linear mixed-effects model was performed using the total test score as the dependent variable. As fixed effects, factors were used with dummy coding^27^ type of evaluation (ECog [original version] and M-ECog), with the patient and the informant according to the diagnostic criteria. The intercept of the patients was used as a random variable. Statistical analysis was performed in R studio v 4.1.2 using the lme4 package for the regressive model and the emmeans package for *post-hoc* analyses. A p-value <0.05 was considered statistically significant.

### Ethics statement

The study protocol was approved by the Institutional Ethics Committee (GER-4153-22-22-1). The study was conducted in accordance with the guidelines of the Declaration of Helsinki and Good Clinical Practice. The appropriate signed consent form was obtained for patients and informants of each of the four groups prior to study participation. Guidelines were used for the process of cross-cultural adaptation of self-report measures^28,29^.

## RESULTS

### Characteristics of demographic variables

Two hundred participants and 200 informants are included in the main analyses. The dementia group was the oldest 77.9±9.8 (p<0.001). Sixty-three percent of the sample were women (p=0.22). Educational level in the CH group was 14.4±6.3 years, in SCD it was 11.9±5.1 and, in dementia, 9.3±4.9 years (p<0.001). Regarding the informants’ characteristics, the mean age was 47.2±5.8 years, 73% were women (p=0.012) and the mean schooling was 10.8±4.6 years. (p=0.93). No significant differences were observed in the Zarit (caregiver collapse) assessment. The mean MMSE was 28.8±1.1 in CH, 27.6±1.5 in SCD, 26.2±1.9 in DCL, and 21.9±3.3 points in dementia (p<0.001). The overall cognitive performance by MoCA-E was 28±1.1 in the CH group, 26.1±2.2 with SCD, 21.3±2.6 with MCI and 15.6±3.3 points with dementia (p<0.001). Global cognitive means by M-ECog were statistically significant between groups, the higher score for degree of cognitive impairment being 43.5±2.6 in CH, 52.7±5.4 in the SCD group, 67.4±9.8 in the MCI group and 85.2±26.2 in the dementia group (p<0.001) (Table 1).

**Table 1.** Sociodemographic characteristics of the participants and informants.

Participants	CH	SCD	MCI	Dementia	p
	n=50	n=50	n=50	n=50	
Age (years), Mean (SD)	70.1±7.7	72.32±7.96^a^	73.3±6.9^b^	77.9±9.8^c^	<0.001
Women (n)%	28 (56)	34 (68)	36 (72)	28 (56)	0.02
Marital status (n)%					
Married	28 (56)	21 (42)	24 (48)	26 (52)	<0.001
Single	9 (18)	11 (22)	8 (16)	3 (6)	<0.001
Divorced	2 (4)	4 (8)	4 (8)	5 (10)	<0.001
Widower	9 (18)	12 (24)	14 (28)	16 (32)	<0.001
Education, mean (SD)	14.4±6.3	11.9±5.1	10.4 ±4.8	9.30 ±4.9	<0.001
MMSE, mean (SD)	28.8±1.1	27.6±1.5	26.2±1.9	21.9±3.3	<0.001
MoCA-E, mean (SD)	28.0±1.1	26.1±2.2	21.3±2.6	15.6±3.3	<0.001
Katz, mean (SD)	6±0.0	6±0.0	6±0.5	5±0.8	<0.001
Lawton & Brody, mean(SD)	8±0.0	8±0.0	7.7±0.5	5±1.5	<0.001
M-ECog, mean (SD)	43.5±2.6	52.7±5.4	67.4±9.8	85.2±26.2	<0.001
Memory, mean (SD)	10.6±1.5	13.1±3.3	16.8±3.4	20.5 ±6.1	<0.001
Language, mean (SD)	9.4±1.0	13.4±2.6	14.7±3.7	18.9±6.8	<0.001
Visuospatial, mean (SD)	7.8±1.0	8.2±1.6	10.1±2.7	13.3±6.9	<0.001
Planning, mean (SD)	5.2±0.6	5.7±1.2	7.1±2.5	10.7±4.2	<0.001
Organization, mean (SD)	4.9±1.2	6.9± 1.2	8.5±2.5	12.6±4.4	<0.001
Divided attention, mean (SD)	5.9±0.7	6.6±2.3	8.5±2.9	9.8±3.8	<0.001
Informants	n=50	n=50	n=50	n=50	
Age (years), mean (SD)	53.7±16.5	53.4±16.8^a^	53.4 ±16.3^b^	53.7±14.9^c^	<0.001
Woman, (n)%	34 (68)	35 (70)	34 (68)	43 (86)	0.12
Marital status (n)%					
Married	24 (48)	13 (41.9)	27 (54)	28 (56)	<0.001
Single	18 (36)	12 (38.7)	19 (38)	17 (34)	<0.001
Divorced	4 (8)	3 (3.7)	2 (4)	2 (4)	<0.001
Widower	2 (4)	2 (6.5)	2 (4)	1 (2)	<0.001
Education, mean (SD)	13.6±4.5	13.9±3.9	13.3±5.1	13.70±3.9	0.93
Caregiver’s burden	5.5±8.6	11.8± 11.7	14.18±14.8	24±13.0	0.16
M-ECog, mean (SD)	45.1±8	54.4±12.9^a^	68.3±13.8^b^	94.4±25.3^c^	<0.001
Memory, mean (SD)	11.0 ±2.4	14.1±3.1	17.7±4.9	23.7±6.6	<0.001
Language, mean (SD)	9.6±2.3	12.1±3.8	14.6±4.4	19.4±6.5	<0.001
Visuospatial, mean (SD)	7.9±1.6	9.1±2.7	11.2±3.7	14.5±6.8	<0.001
Planning, mean (SD)	5.2±0.8	5.9±1.9	8.2±2.8	11.5±5.3	<0.001
Organization, mean (SD)	6.3±1.5	7.2±2.6	9.1±3.0	15.2±5.4	<0.001
Divided attention, mean (SD)	5.4±1.6	6.1±2.5	7.8±2.6	10.4±3.3	<0.001

Abbreviations: CH, cognitively healthy; SCD, subjective cognitive decline; MCI, mild cognitive impairment; SD, standard deviation. Notes: ^a,b,c^Post-hoc comparisons, Bonferroni correction; ^a^p≤0.001 between CH and SCD; ^b^p≤0.001 between CH and MCI; ^c^p≤0.001 between CH and dementia.

### Cross-cultural adaptation

**Figure 1.**
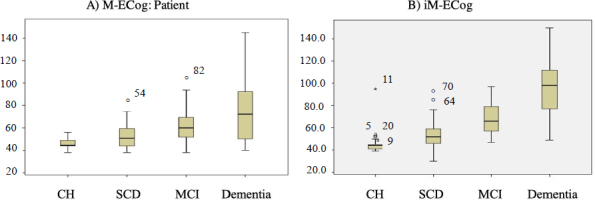
Box and whisker plot of Everyday Cognition Scale Mexican version partipants and informants. A: Everyday Cognition Scale Mexican version: Patients; B: Everyday Cognition Scale Mexican version: Informants.

Abbreviations: M-ECog, Everyday Cognition Scale Mexican version; CH, cognitively healthy; SCD, subjective cognitive decline; MCI, mild cognitive impairment.

In the mixed-effects linear model of the ECog (original version and M-ECog), the diagnostic factor was significant for all groups in the original version ECog (p<0.002), indicating that all groups had significantly higher scores compared to the control group (Figure 1). In addition, a significant interaction between the patient and informant ECog was found in the dementia group (t-value -6.382, p<0.001), revealing that informants tend to rate patients with dementia higher compared to the patient’s self-assessment (p<0.001) (Supplementary Material 2, Table A1). *Post-hoc* analyses of the three-way interaction showed that M-ECog scores in the dementia group were higher than those of the original ECog, both in the self-assessment (β=-7.17, p=0.04 ) and in that carried out by the informants (β=-23.28, p<0.001). No other factors or interactions reached statistical significance (p=0.13).

### Validity

Construct validity when comparing with the global punctuation between M-ECog *vs*. MMSE and M-ECog *vs*. MoCA-E (Spearman correlation test) was 0.681 (p<0.001). The global and specific scores for each of the 6 M-ECog domains were statistically significantly correlated with MMSE, MoCA-E, and ADLs and IADLs activities (Katz, Lawton & Brody) (Table 2).

**Table 2.** Characteristics of domain subscores by cognitive group between Everyday Cognition Scale Mexican version and Mini-Mental State Evaluation, Montreal Cognitive Assessment Spanish version and activities of daily living.

M-ECog		CH	SCD	MCI	Dementia	Correlation Spearman	p
MMSE, mean, (SD)	Global cognition	28.8±1.1	27.6±1.5	26.2±1.9	21.9±3.3	-0.635	<0.001*
	Memory	3.0±0.0	3.0±0.0	3.0±0.0	2.9±0.4	-0.244	0.006
	Language	2.0±0.0	2.0±0.0	2.0±0.0	1.9±0.2	-0.660	0.464
	Visuospatial	0.62±0.5	0.4±0.5	0.3±0.5	0.1±0.3	-0.266	0.003
	Attention	4.82±0.38	4.82±0.40	4.4±0.9	3.7±1.5	-0.224	0.120
MoCA-E, mean, (SD)	Global cognition	28.0±1.1	26.1±2.2	21.3±2.6	15.6±3.3	-0.701	<0.001*
	Memory	4.1±0.8	3.4±1.3	2.0±1.8	1.2±1.3	-0.761	<0.001
	Language	2.9±0.3	2.7±0.6	2.0±0.9	0.9±0.9	-0.308	<0.001
	Visuospatial	4.2±0.73	3.5±0.9	3.2±4.4	1.7±1.1	-0.264	0.003
	Planning	13.6±1.5	12.2±2.9	9.3±4.1	7.4±3.4	-0.273	0.002
	Organization	2.9±0.2	2.9±0.3	2.7±0.5	2.4±0.7	-0.211	0.017
	Attention	5.7±0.6	5.4±0.9	4.3±1.1	2.9±1.4	-0.267	0.002
ADLs (Katz) mean, (SD)	Global function	6±0.0	6±0.0	6.0±0.5	5.±0.8	-0.383	<.0001
	Memory	6±0.0	6±0.0	5.9±0.2	5±0.8	-0.353	<0.001
	Language	6±0.0	6±0.0	5.9±0.2	5±0.8	-0.362	<0.001
	Visuospatial	6±0.0	6±0.0	5.9±0.2	5±0.8	-0.102	0.252
	Planning	6±0.0	6±0.0	5.9±0.2	5±0.8	-0.299	0.001
	Organization	6±0.0	6±0.0	5.9±0.2	5±0.8	-0.393	<0.001
	Attention	6±0.0	6±0.0	5.9±0.2	5±0.8	-0.284	0.001
ADLs (Lawton & Brody) mean, (SD)	Global function	8±0.0	8±0.0	7.7±0.5	5±1.5	-0.409	<0.001
	Memory	8±0.0	8±0.0	7.5±0.5	4.5±1.5	-0.404	<0.001
	Language	8±0.0	8±0.0	7.5±0.5	4.5±1.5	-0.364	<0.001
	Visuospatial	8±0.0	8±0.0	7.5±0.5	4.5±1.5	-0.152	0.086
	Planning	8±0.0	8±0.0	7.5±0.5	4.5±1.5	-0.407	<0.001
	Organization	8±0.0	8±0.0	7.5±0.5	4.5±1.5	-0.456	<0.001
	Attention	8±0.0	8±0.0	7.5±0.5	4.5±1.5	-0.249	0.005

Abbreviations: M-ECog, Everyday Cognition Scale Mexican version; MMSE, Mini-Mental State Evaluation; SD, standard deviation; MoCA-E, Montreal Cognitive Assessment Spanish version; ADLs, activities of daily living; CH, cognitively healthy; SCD, subjective cognitive decline; MCI, mild cognitive impairment.

### Reliability

The internal consistency of M-ECog estimated to Cronbach’s alpha index was 0.881. The ICC was 0.877 (95% confidence interval — 95%CI 0.850–0.902; p<0.001). M-ECog was significantly correlated with ADLs (0.40) ([95%CI 0.320–0.471; p<0.001]), MMSE (0.68) ([95%CI 0.650–0.710; p<0.001]) and MoCA-E (0.70) ([95%CI 0.620–0.892; p<0.001]).

**Figure 2.**
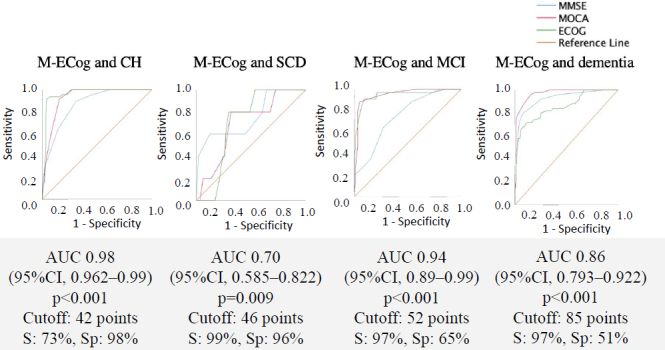
Receiver operating characteristic curves patient group.

Abbreviations: M-ECog, Everyday Cognition Scale Mexican version; MMSE, Mini-Mental State Evaluation; MoCA, Montreal Cognitive Assessment; CH, cognitively healthy; SCD, subjective cognitive decline; MCI, mild cognitive impairment; ECog, Everyday Cognition Scale; AUC, area under the ROC curve; 95% CI, 95% confidence interval; S, sensitivity; Sp, specificity.

The results of the sensitivity, specificity, and AUC of M-ECog participant group were the following: in CH, the AUC of M-ECog was 0.98 (95%CI 0.96–0.99, p<0.001) with a cut-off value of 42 points, and S: 73%, Sp: 98%, PPV: 0.97 and NPV: 0.43. For SCD, the AUC of M-ECog was 0.70 (95%CI 0.58–0.82, p<0.005) with a cut-off value of 46 points, and S: 99%, Sp: 96%, PPV: 0.83, NPV: 0.88. For MCI, the AUC of M-ECog was 0.94 (95%CI 0.89–0.99, p<0.001) with a cut-off value of 52 points, and S: 97%, Sp: 65%, PPV: 0.87, NPV: 0.47. For the dementia group, the AUC of M-ECog was 0.86 (95%CI 0.79–0.92, p<0.001) with a cutoff value of 85 points, and S:75%, Sp 74%, PPV: 0.92, NPV 0.50 (Figure 2).

**Figure 3.**
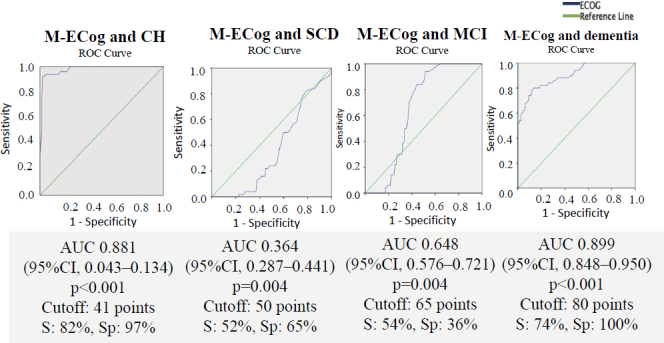
Receiver operating characteristic curves informant group.

Abbreviations: ECog, Everyday Cognition Scale; CH, cognitively healthy; SCD, subjective cognitive decline; MCI, mild cognitive impairment; AUC, area under the ROC curve; 95%CI, 95% confidence interval; S, sensitivity; Sp, specificity.

Regarding the M-ECog, mean scores by groups were: CH, 45.1±8; SCD, 54.4±12.9; MCI, 68.3±13.8; and dementia, 94.4±25.1 points (p<0.001). The ROC curves for the informant group are shown in Figure 3, which highlights that the AUC of the dementia group was 0.899 ([95%CI 0.848–0.950] p<0.001), with a cut-off value of 80 points (S: 74%, Sp:100%).

The cut-off values for M-ECog between the different groups were: 45 points for CH (S: 78%, Sp: 99%, PPV: 0.97, NPV: 0.45); 55 points for SCD (S: 96%, Sp: 83%, PPV: 0.83, NPV: 0.88); 75 points for MCI (S: 96%, Sp: 42%, PPV: 0.87, NPV: 0.47); and a score greater than 80 points for the dementia group (S: 75%; Sp: 74%; VVP: 0.73; NPV: 0.25).

## DISCUSSION

Since its creation, ECog was developed to assess functional capacity that is clearly linked to specific cognitive abilities^30^. It has been validated in multiple studies to identify subjects with MCI^6^ and mild to moderate dementia^13^. In recent years it has also been beneficial for SCD screening in older people^31^. This has become highly relevant because this condition is recognized as a risk state for developing MCI (annual conversion rate 6.67% [95%CI 4.70–8.95%]) and dementia (relative risk — RR 2.07 [95%CI 1.76–2.44]) when compared to cognitively healthy individuals^32^.

In our study, we show that M-ECog is a valid and reliable scale for screening SCD, MCI, and dementia in older Mexican adults. This cross-cultural adaptation allowed showing an adequate correlation between the information obtained from the patient and the informant. Results of previous studies have shown that the Spanish version of ECog is useful to evaluate participants with MCI and Alzheimer’s disease (Cronbach’s alpha 0.98)^15^. Thus, M-ECog as applied to the Mexican population showed the ability to discriminate between different cognitive declines (Cronbach’s alpha was 0.881).

In addition, it has been possible to establish cut-off points for the different clinical conditions proposed in the analysis of this study: for CH 45 points (S: 78%, Sp: 99, PPV: 0.97, NPV: 0.45), for SCD 55 points (S: 96%, Sp: 83%, PVP: 0.83, NPV 0.88), for MCI 75 points (S: 96%, Sp:42%, VPP:0.87, NPV 0.47), and for dementia over 80 points (S: 75%, Sp: 74%, VVP: 0.73, NPV: 0.25). It is known that the scale has been designed so that a higher score represents worse cognitive performance, and among its strengths are the possibility of self-application and not requiring extensive training and prior standardization for its application^25^. Patients and informants can question what helps diagnostic accuracy, especially in the early stages of cognitive impairment. In this study, the CH, SCD and MCI groups reported problems in daily function in the M-ECog according to the informants, while in dementia it was observed that the patients self-reported less functional impairment in comparison to the qualifications of their informants. This would be associated with the course of the disease itself and the possible anosognosia frequently reported in patients with cognitive impairment^33^. However, we believe that this issue needs further investigation.

Screening people with cognitive impairment continues to be a challenge in clinical practice^34^. The systematic use of scales that measure the impact of cognition on functionality has shown utility^30^ by providing information that is relevant to stratify the severity of the deterioration, as well as to provide practical recommendations to patients who require supervision in certain cognitive areas and continuous support from family members or social service^15^. Therefore, this instrument opens the possibility of comprehensive and early cognitive screening, since it is known that SCD can be masked by concomitant conditions such as depression, anxiety, sensory deficit^35^, and post-surgical delirium^36^. Therefore, its early identification would allow timely interventions that would reduce the loss of autonomy of the elderly and the impact of related public health costs^37^.

The strengths of this study are several:

- It allowed the cross-cultural validation of the M-ECog instrument with an ICC of 0.877 (p<0.001);
- The M-ECog can be used in Spanish-speaking countries;
- The M-ECog is sensitive and specific to discriminate cognitive impairment from early stages such as SCD and MCI to late stages such as dementia;
- Cut-off points are proposed for each of the four cognitive groups. Some of the limitations of our study are the sample size and that our sample is only from one city in Mexico, so that it might not be representative of the national population of older adults in Mexico. The city is a highly urban area, resulting in a group with higher education than the overall Mexican population of older adults, and this can result in a selection bias. Therefore, it is necessary that this validated instrument be used in other studies to know its external validation, and given the level of education, the cut-off point reported in our study should continue to be analyzed in groups with different levels of education in the country itself. Lastly, another limitation is that data on amyloid and tau biomarkers and neuroimaging were not included in this study.

In conclusion, the M-ECog scale proves to be valid and reliable for measuring everyday abilities mediated by cognition. It is self-applicable (patient and informant) without requiring extensive prior formation, which makes it practical and extrapolated to various clinical settings. Our results showed that the M-ECog scale had high accuracy, exhibiting a high percentage of correct classification compared to the clinical diagnosis, and it is useful to screen for SCD and MCI in older Mexican adults.
